# The effect of EGFR‐TKIs on survival in advanced non‐small‐cell lung cancer with EGFR mutations: A real‐world study

**DOI:** 10.1002/cam4.5413

**Published:** 2022-11-15

**Authors:** Xiaoqing Yu, Jinfei Si, Jingwen Wei, Yanling Wang, Yan Sun, Jianan Jin, Xiaoyan Zhang, Tonghui Ma, Zhengbo Song

**Affiliations:** ^1^ Department of Clinical Trial The Cancer Hospital of the University of Chinese Academy of Sciences (Zhejiang Cancer Hospital) Hangzhou China; ^2^ Institute of Basic Medicine and Cancer (IBMC), Chinese Academy of Sciences Hangzhou China; ^3^ Department of Oncology The Second Clinical Medical College of Zhejiang Chinese Medical University Hangzhou China; ^4^ Department of Oncology The First Clinical Medical College of Wenzhou Medical University Wenzhou Zhejiang China; ^5^ Department of Translational Medicine, Genetron Health (Beijing) Technology, Co. Ltd. Beijing China

**Keywords:** EGFR‐TKIs, non‐small cell lung cancer (NSCLC), real‐world study, survival outcome

## Abstract

**Background:**

Few large‐scale studies have been published using real‐world data related to overall survival (OS) improvements in advanced epidermal growth factor receptor (EGFR)‐mutant lung cancer patients; therefore, little is known regarding the characteristics of patients who could benefit most from EGFR‐tyrosine kinase inhibitors (TKIs). Our study aimed to assess whether EGFR‐TKI treatment confers survival benefits among advanced non‐small‐cell lung cancer (NSCLC) patients harboring EGFR mutations in the Chinese population.

**Patients and Methods:**

A total of 6451 advanced NSCLC patients were diagnosed between January 1, 2013 and June 30, 2019 in Zhejiang Cancer Hospital. Ultimately, 2864 patients with a confirmed EGFR mutation genotype were enrolled in our study. OS was measured from the time of diagnosis of advanced NSCLC until death or last follow‐up.

**Results:**

Median follow‐up for OS of advanced EGFR‐mutant NSCLC patients was 28.33 months in our study. Patients who received EGFR‐TKIs demonstrated better survival compared to those without EGFR‐TKI treatment (mOS: 29.77 vs. 22.97 months, *p* < 0.0001). A total of 451 patients switched to third‐generation EGFR‐TKI treatment and obtained a significantly better survival than those who adopted first‐line third‐generation EGFR‐TKIs or those who did not receive third‐generation EGFR‐TKIs after disease progression with first‐ or second‐generation EGFR‐TKI treatment (mOS: 38.0 vs. 32.5 vs. 28.3 months, *p* < 0.0001). As for EGFR genotypes, patients with exon 19 deletion showed better OS, followed by those with L858R mutation (32.4 vs. 24.83 months, *p* = 0.0013). NGS versus PCR testing showed no statistical differences with respect to survival outcomes (mOS: 27.5 vs. 27.47 months, *p* = 0.6745).

**Conclusion:**

Advanced EGFR‐mutant patients treated with EGFR‐TKIs obtained absolute superior survival in the Chinese population.

## 1 INTRODUCTION

Lung cancer is a leading cause of mortality worldwide.^1^ It is reported that, with the approvals for and use of targeted therapies since 2013, population‐level mortality from non‐small‐cell lung cancer (NSCLC) in the United States decreased 6.3% annually from 2013 to 2016, and survival after diagnosis improved to 35%.^2^ The longest overall survival (OS) of advanced nonsquamous NSCLC patients treated with chemotherapy was reported to be 24.3 months, according to the BEYOND study.^3^

Asian patients with adenocarcinoma have a higher epidermal growth factor receptor (EGFR) mutation frequency of about 51.4% overall.^4^ Since 2009, the IPASS study^5^ first prospectively demonstrated the clinical efficacy of EGFR‐ tyrosine kinase inhibitors (TKIs) in patients with advanced NSCLC harboring EGFR mutations. This marked the beginning of the era of precision medicine of lung cancer. Several subsequent phase III trials have confirmed that first‐ and second‐generation EGFR‐TKIs are superior to chemotherapy in terms of progression‐free survival (PFS) and objective response rates (ORR),^6^, ^7^, ^8^, ^9^, ^10^ but no improvement was observed in OS (range, 24.5–34.1 months).^11^, ^12^, ^13^

Currently, the third‐generation EGFR‐TKI, osimertinib, is making another breakthrough in lung cancer targeted therapy. According to the FLAURA study, it not only prolongs PFS but also improves OS as well^14^, ^15^; patients treated with osimertinib have shown an OS of 38.6 months compared to an OS of 31.8 months in the comparator group (HR = 0.80; 95.05% CI, 0.64 to 1.00; *p* = 0.046). However, very few members of the Chinese population were enrolled in the FLAURA study. Therefore, the results of Asian subgroup analysis are not representative of the Chinese population. It is expected that the results of this study will bring more evidence for use of Osimertinib as a first‐line treatment in Chinese NSCLC patients with EGFR mutations. Additionally, the FLAURA study did not compare survival of EGFR wild type patients. In order to further explore the benefits of osimertinib in the Chinese population, the researchers carried out the FLAURA China study.^16^ The study enrolled only 136 Chinese patients, and the results showed median OS of 33.1 months in the osimertinib group versus 25.7 months in the comparator group (HR = 0.85; 95% CI 0.56–1.29).

The interim analysis of the FLOURISH study^17^ was presented at the 2022 ESMO conference. The FLOURISH study explored real‐world outcomes of first‐line osimertinib for EGFR mutated advanced NSCLC patients in China. After a median follow‐up of 10.2 months, the 1‐year PFS rate was 78.8%. In addition, the final analysis of the ASTRIS China subgroup was announced at the 2022 World Lung Cancer Congress (WCLC).^18^ The result showed that osimertinib could obtain PFS benefit after the resistance of first‐line EGFR‐TKIs in a real‐world Chinese population. The ASTRIS study further confirmed the results of AURA3 and consolidated the status of standard treatment in patients with secondary T790M mutation.

Few large‐scale studies have been published using real‐world data with respect to OS improvements in advanced NSCLC patients harboring EGFR mutations, and specifically the characteristics of patients who could benefit most from EGFR‐TKIs. In China, due to medical insurance limitations, EGFR‐TKIs (gefitinib, erlotinib, and icotinib) were not covered by Medicare until 2016. Since then, an increasing number of patients with EGFR mutations were eligible for treatment with EGFR‐TKIs. With such a large group of patients only recently receiving EGFR‐TKI treatment, the influence of EGFR‐TKIs on overall survival has not been well described. Therefore, we aimed to determine whether patients harboring EGFR mutations achieve survival benefits from EGFR‐TKI treatment. To address this, we carried out this study aiming to assess whether EGFR mutation is correlated with differences in survival among advanced NSCLC patients harboring EGFR mutations in the Chinese population. We also aimed to investigate the survival differences resulting from diverse EGFR treatment approaches in real‐world practice.

## 2 PATIENTS AND METHODS

### 2.1 Clinical data

This retrospective cohort study used the database of Zhejiang Cancer Hospital. A total of 6451 advanced NSCLC patients were diagnosed between January 1, 2013 and June 30, 2019. Among them, patients who met the following criteria were enrolled in this study: (a) histologically or cytologically confirmed pulmonary malignancy; (b) confirmed EGFR mutation genotype; (c) age 18 years or older; and (d) availability of complete follow‐up records. Finally, 2864 patients with confirmed EGFR mutation genotype were ultimately enrolled in our study. OS was defined as time from diagnosis of advanced NSCLC until death or the last telephone follow‐up. EGFR status was tested by next‐generation sequencing (NGS) or polymerase chain reaction (PCR). The NGS was College of American Pathologist (CAP)‐accredited and panels varied, but all of them included the nine genes mentioned as molecular biomarkers in the National Comprehensive Cancer Network guidelines (EGFR, ALK, ROS1, KRAS, HER2, BRAF, MET, RET, NTRK). A propensity score‐matched survival analysis was used to evaluate differences in OS between different subgroups.

### 2.2 Data source

The Database of Zhejiang Cancer Hospital included deidentified patient data that were obtained under an institutional review board‐approved protocol with a waiver of informed consent. The Investigations Committee of Zhejiang Cancer Hospital determined that this study did not include any human subjects research. Written informed consent was waived by the ethical committee. Both structured and unstructured data were obtained. The database represents a geographically and demographically diverse population of patients with cancer, along with information about clinicians and health care institutions. All datasets included demographic data, such as age and sex, as well as clinical data, such as performance status, pathological type, EGFR mutation type, and treatments. Tumor genomic testing results were obtained from the medical records of the patients and time of death was abstracted via telephone follow‐up.

### 2.3 Statistical analysis

The primary endpoint was OS. The OS and prognostic factors were evaluated in univariate and multivariate analyses. OS was estimated by the Kaplan–Meier curve and the differences were tested by log‐rank test with the hazard ratios calculated using the Mantel–Haenszel method. A two‐tailed *p*‐value of <0.05 was considered statistically significant. Statistical analyses were performed using GraphPad Prism 6 (version 6, GraphPad Software San Diego, CA, USA) and SPSS 22.0 (SPSS, Chicago, IL, USA).

## 3 RESULTS

### 3.1 Patients

Baseline patient clinical characteristics are provided in Table 1. The median age was 52 years, ranging from 26 to 86 years. A total of 1544 patients (53.9%) were older than 60 years, 1261 patients (44.0%) were male, 2602 patients (90.9%) had confirmed histology of adenocarcinoma, 1945 patients (67.9%) were non‐smokers (71 patients had unknown smoking status), 2262 patients (79.0%) were stage IV at diagnosis, and 1410 patients (52.5%) had no comorbidities. Among the 2864 total patients, 2459 (85.9%) had received EGFT‐TKIs; 33 had received a third‐generation EGFR‐TKI as first‐line treatment, and 410 (14.3%) had switched to a third‐generation EGFR‐TKI following disease progression after treatment with first‐ or second‐generation EGFR‐TKIs. A total of 1312 patients (45.8%) harbored EGFR 19del mutations and 1337 patients (46.7%) had L858R mutation (additional details are provided in Table 1). There were 110 patients (3.8%) harboring multiple mutations and 46 patients (1.6%) harboring uncommon mutations. For most patients (85.5%), PCR was used to detect EGFR status; NGS was used in only 415 patients (14.5%).

**TABLE 1:** Baseline of patient and treatment characteristics

	All (*n* = 2864)
**Male, *n* (%)**	1261 (44.0%)
**Age, Mean (SD)**	52, 26–86
**Histopathology, *n* (%)**	
Adenocarcinoma	2602 (90.9%)
Others	
**Smoking status, *n* (%)**	
Non‐smoker	1945 (67.9%)
**Comorbidity, *n* (%)**	
0	1410 (52.5%)
1–2	1356 (47.3%)
>3	98(3.4%)
**Year of diagnosis, n (%)**	
2013–2014	229 (8.0%)
2014–2015	325 (11.3%)
2015–2016	449 (15.7%)
2016–2017	582 (20.3%)
2017–2018	586 (20.5%)
2018–2019	693 (24.2%)
**Stage at diagnosis, *n* (%)**	
IV	2262(79.0%)
**Treatments, *n* (%)**	
EGFR‐TKIs	2459 (85.9%)
1G‐3G	410 (14.3%)
Others	405 (14.1%)
**EGFR mutation, *n* (%)**	
exon 18	27 (0.9%)
exon 19	1317 (46.0%)
19del	1312 (45.8%)
exon 20	150 (5.2%)
exon 21	1370 (47.8%)
L858R	1337 (46.7%)
**Mutation status, *n* (%)**	
Single EGFR mutation	2754 (96.2%)
With multiple mutations	110 (3.8%)
With uncommon mutations	46 (1.6%)
**Genetic testing, *n* (%)**	
NGS	415 (14.5%)
Routine	2449 (85.5%)

### 3.2 Overall survival

**FIGURE 1:**
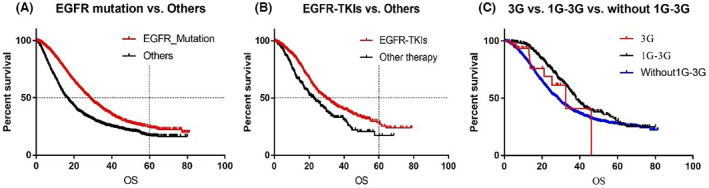
Kaplan–Meier curve illustrating the overall survival (OS) of epidermal growth factor receptor (EGFR)‐mutant non‐small‐cell lung cancer (NSCLC) patients. (A) EGFR mutation versus others. (B) EGFR‐targeted therapy versus other therapy. (C) 3G versus 1G‐3G versus without 1G‐3G

Median follow‐up for OS (mOS) was better for patients harboring EGFR mutations compared to those without EGFR mutation (mOS: 28.33 vs. 16.4 months, *p* < 0.0001; Figure 1A) and the 5‐year OS rate was also improved (24.5% vs. 17.2%, respectively). In addition, patients who received EGFR‐TKIs demonstrated better survival compared to those without EGFR‐TKIs (mOS: 29.8 months vs. 23.0 months, *p* < 0.0001; Figure 1B) and the 5‐year OS rate reached 28.4% and 17.2%, respectively. In addition, 410 patients switched to third‐generation EGFR‐TKI and achieved significantly better survival than those who adopted first‐line third‐generation EGFR‐TKIs (33 patients) or those who did not receive third‐generation EGFR‐TKIs after disease progression with first‐ or second‐generation EGFR‐TKI treatment (2016 patients) (mOS: 38.0 vs. 32.5 vs. 28.3 months, *p* < 0.0001; Figure 1C). We further used a propensity score‐matched survival analysis to evaluate differences in overall survival between patients with or without ICIs. The application of ICIs after the resistance of EGFR‐TKIs had no statistical difference contributing to survival outcomes (*p* = 0.504).

Among patients who received EGFR‐TKIs, we included sex, age, comorbidity, smoking status, and stage IV diagnosis as independent pretreatment prognostic factors in the Cox regression model. In our analysis, stage IV diagnosis was an independent prognostic factor.

### 3.3 EGFR genotypes

**FIGURE 2:**
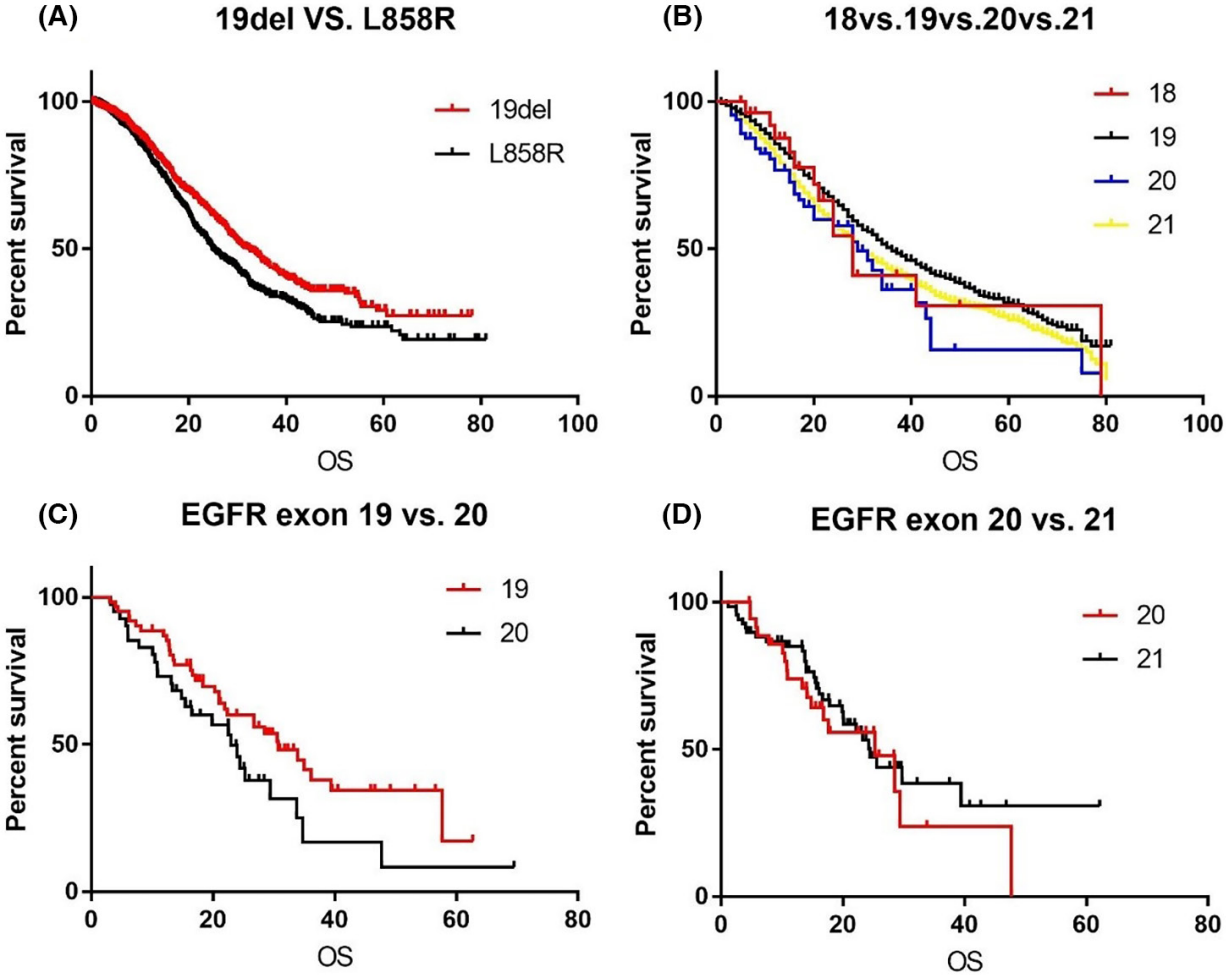
Kaplan–Meier curve illustrating the OS of different EGFR genotypes. (A) EGFR 19del versus L858R. (B) EGFR exon 18 versus exon 19 versus exon 20 versus exon 21. (C) EGFR exon 19 versus exon 20. (D) EGFR exon 20 versus exon 21

As for EGFR genotypes, the results of our study showed significant survival differences between patients with EGFR 19 deletion and L858R mutation (32.4 months vs. 24.8 months, *p* = 0.0013; Figure 2A). In this study, we defined all EGFR genotypes except EGFR 19 deletion and L8585R as uncommon mutations, including exon 18 mutations (G719X, E709X, R776C), exon 20 mutations (20ins, S768I, T790M, G779C), and exon 21 mutations (L861Q, L861R, G863C). We further analyzed the survival differences between different EGFR subtypes, and found that exon 18 mutations and exon 20 mutations had poor survival outcomes (exon 18 vs. exon 19 vs. exon 20 vs. exon 21 = 28.0 vs. 36.0 vs. 29.0 vs. 30.0, *p* = 0.0006, Figure 2B; exon 19 vs. exon 20 = 30.7 vs. 23.0, *p* = 0.0510, Figure 2C; exon 20 vs. exon 21 = 25.3 vs. 24.5, *p* = 0.5169, Figure 2D; exon 18 vs. exon 20 = 14.5 vs. undefined, *p* = 0.4635, Figure S1).

In addition, patients harboring single EGFR mutations showed no statistically significant differences in survival compared to patients harboring multiple mutations (25.6 months vs. 25.3 months, *p* = 0.6475; Figure S2). However, in patients with multiple mutations, we found that patients with uncommon mutations have poorer OS (*p* = 0.0003, Supplementary Figure 3).

### 3.4 NGS versus PCR

**FIGURE 3:**
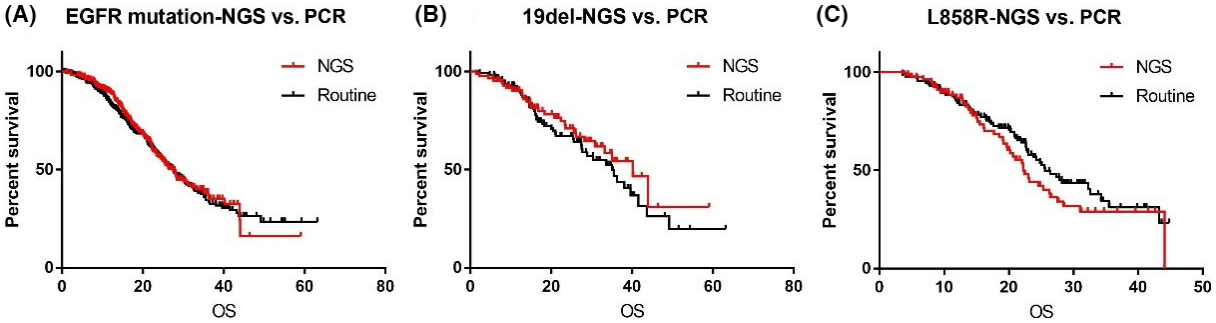
Kaplan–Meier curve illustrating the OS of different detection methods for EGFR mutations. (A) NGS versus PCR for EGFR mutations. (B) NGS versus PCR for EGFR 19del. (C) NGS versus PCR for EGFR L858R

In the comparison of detection methods for EGFR mutation, NGS and PCR showed no statistical differences contributing to survival outcomes (27.5 months for NGS vs. 27.47 months for PCR, *p* = 0.6745; Figure 3A). Median OS for the subgroup with EGFR 19 deletion also showed no statistical difference (40.1 months for NGS vs. 35.0 months for PCR, *p* = 0.3446; Figure 3B) nor did the L858R subgroup. There was a tendency for poorer survival with NGS detection subgroup (22.23 months for NGS vs. 25.6 months for PCR, *p* = 0.1835; Figure 3C).

## 4 DISCUSSION

Outside of clinical studies, there are limited data related to improved survival outcomes in advanced EGFR‐mutant patients. Our study demonstrated that EGFR‐TKIs are beneficial to the survival of Chinese patients. As far as we know, this is the first large‐scale study addressing EGFR‐mutant patients who were prescribed EGFR‐TKIs in a real‐world clinical setting in China, and our findings can be of guiding significance to clinical practice.

In our analysis, patients harboring EGFR mutations showed superior survival outcomes (OS = 28.3 months) compared to those without EGFR mutations. Advanced EGFR‐mutant patients treated with EGFR‐TKIs obtained absolute superior survival compared to those receiving other treatments, including chemotherapy, anti‐vascular endothelial growth factor (anti‐VEGF), and immunotherapy (29.8 months for EGFR‐TKIs vs. 23.0 months for others). Our results are consistent with several completed phase III trials of first‐line EGFR‐TKIs versus chemotherapy,^7^, ^8^, ^9^, ^10^, ^11^, ^15^ ranging from 18.8 to 30.5 months; osimertinib has data points reaching 38.6 months. Bergqvist previously reported on real‐world survival data for Swedish patient from 2010 to 2016.^19^ The median OS was 15.5 months and longer OS was achieved with more recent diagnosis (18.6 months in years 2014–2015). In view of the differences in mutation spectrum between Eastern and Western populations, it seems EGFR‐TKIs achieve better survival outcomes in Chinese patients. Additionally, patients with EGFR mutations are sensitive to anti‐VEGF agents.

Due to medical insurance limitations, osimertinib was not covered by Medicare as a first‐line treatment until 2021, and only 33 patients enrolled in our study adopted first‐line osimertinib treatment. Even so, we found patients who had switched to third‐generation EGFR‐TKIs after disease progression with first‐ or second‐generation EGFR‐TKI treatment showed the best survival benefits compared to those who adopted first‐line third‐generation EGFR‐TKIs or those who did not receive third‐generation EGFR‐TKIs after progression. Almost 60% of patients acquired the T790M mutation after progression on TKIs^20^ and osimertinib was the standard of care for these patients. According to the previous AURA series studies,^21^, ^22^, ^23^ osimertinib significantly prolonged the PFS of patients with acquired T790M mutations. Osimertinib also demonstrated great efficacy in patients with T790M‐positive advanced NSCLC who experienced disease progression with first‐line EGFR‐TKI therapy. The FLAURA study subsequently adopted osimertinib as a first‐line treatment for advanced EGFR‐mutant patients, updated the survival outcomes of all previous first‐line treatments,^14^ and recently reported an OS of 38.6 months.^15^ In our study, patients who switched to third‐generation EGFR‐TKIs showed better survival than those who adopted first‐line third‐generation EGFR‐TKIs. The OS was slightly shorter than the reported 38.6 months of the FLAURA study, but was similar that reported for the Asian subgroup. Meanwhile, the physical condition of patients in real‐world studies is generally inferior to patients in clinical trials, which may also explain this difference.

Among the various EGFR mutation subtypes, including exon 19 deletion, 21 L858R, G719X, L861Q, and other uncommon mutations, we found exon 19 deletion achieved better OS, followed by L858R; exon 18 and 20 mutations (20ins/T790M/S768I) had poor survival. A number of studies have reported that the efficacies of various EGFR‐TKIs are distinct among different mutant subtypes. Yang et al. first reported a pooled analysis of LUX‐3 and LUX‐6, which showed an unprecedented overall survival benefit with afatinib treatment compared to chemotherapy for the exon 19 deletion, but not for the L858R mutation.^24^ Several subsequent studies also confirmed that EGFR‐TKIs have greater efficacy in patients with exon 19 deletion than in L858R patients.^25^, ^26^, ^27^ In the ARCHER 1050 study, the PFS of the L858R subgroup was less than that of the 19del subgroup, and the PFS benefit of L858R did not translate into an OS benefit.^13^ Similar results were also reported in the FLAURA study.^15^ It has been reported that 19 deletion and L858R mutation have different biological characteristics with respect to genomics and proteomics.^28^, ^29^ A retrospective study reported patients with L858R mutation had a significantly higher incidence of concomitant mutation than those with 19del mutations, which may lead to the inferior survival of L858R patients.^30^ In these uncommon mutation subtypes, patients with exon 18 mutations (mainly G719X) achieve better survival compared to those with exon 20 mutations, but patients with exon 18 mutations fare slightly worse than those with classical EGFR mutations. Yun et al. reported that the affinity of G719S mutations to gefitinib was 50‐fold weaker than that of L858R mutation.^31^ Xu et al. previously reported a real‐world study on EGFR‐TKIs in Chinese patients with uncommon EGFR mutations.^32^ The study showed that L861Q and G719X mutations were correlated with improved survival benefits from EGFR‐TKIs (mPFS: 8.1 and 6.3 months; ORR: 39.6% and 36.8%), whereas 20ins alterations showed limited response to EGFR‐TKIs (mPFS: 2.0 months; ORR: 8.3%). Additionally, many studies have suggested that 20 insertion mutations are not beneficial for EGFR‐TKIs treatment.^33^, ^34^, ^35^, ^36^ In our study, 36% of EGFR exon 20 mutations derived from T790M mutations, which could explain the longer OS observed in the entire exon 20 subgroup.

In our analysis, patients harboring single EGFR mutations showed no statistically significant differences in survival compared to patients harboring multiple mutations. However, in further subgroup analyses, we found patients harboring EGFR mutations coexisting with uncommon mutations derived inferior survival when compared to those without uncommon mutations. It has been reported that compound EGFR mutations comprise 4%–14% of EGFR‐mutant NSCLCs^33^, ^37^, ^38^; although patients with sensitizing mutations in addition to an atypical mutation respond to EGFR‐TKIs, poor efficiency was observed in patients who harbored uncommon EGFR mutations.

In our study, NGS and PCR showed no statistical differences with respect to survival outcomes for patients with EGFR mutations. Presley et al. previously reported that broad‐based genomic sequencing was not associated with better survival than routine EGFR and/or ALK testing among advanced NSCLC patients receiving treatment in the community oncology setting.^39^ We assume most agents for EGFR mutations are already available, thus NGS testing does not show an advantage for patients with EGFR mutations. Thus, for EGFR mutations, PCR can meet the basic demands of clinical use. In our study, both NGS and PCR were reliable in directing the management of EGFR‐TKIs. Interestingly, we found that patients with L858R mutation detected by NGS showed even poorer survival. This may be related to the low abundance detected by NGS, especially since L858R has strong heterogeneity.

A potential limitation of our study is its single‐institutional retrospective design, which may not be representative of the entire Chinese population. Additionally, there may have been bias in selecting patients for enrollment. Lastly, follow‐up data of mid‐term efficacy analysis were incomplete; therefore, we were unable to analyze these factors.

## 5 CONCLUSION

In conclusion, our study demonstrates that advanced NSCLC patients with EGFR mutations in the Chinese population obtained absolute superior survival following treatment with EGFR‐TKIs. Improved survival with EGFR‐TKI treatment was most drastic in patients with EGFR 19 deletion, and treatment with a third‐generation EGFR‐TKI further prolonged survival after disease progression with first‐ or second‐generation EGFR‐TKIs. Additionally, the mutation detection method (NGS vs. PCR) does not seem to have an impact on survival outcome in patients with EGFR mutations.

## AUTHOR CONTRIBUTIONS

**Xiaoqing Yu:** Conceptualization (lead); data curation (equal); writing – original draft (lead); writing – review and editing (equal). **Jinfei Si:** Data curation (equal); formal analysis (equal); methodology (equal); resources (equal). **Jingwen Wei:** Data curation (equal); formal analysis (equal); methodology (equal); resources (equal). **yanling wang:** Formal analysis (equal). **Yan Sun:** Data curation (equal). **Jianan Jin:** Data curation (equal). **Xiaoyan Zhang:** Methodology (equal); software (equal). **Tong‐Hui Ma:** Methodology (equal); software (equal). **Zhengbo Song:** Conceptualization (equal); data curation (equal); formal analysis (equal); investigation (equal); project administration (equal); resources (equal); supervision (equal); writing – review and editing (equal).

## FUNDING INFORMATION

Not applicable.

## CONFLICT OF INTEREST

The authors declare that they have no competing interests.

## ETHICS STATEMENT

The study was approved by the institutional review board of Zhejiang Cancer Hospital (IRB No. 2022–103) and individual consent for this retrospective analysis was waived.

## CONSENT FOR PUBLICATION

Final approval of manuscript: all authors.

## Supporting information


Figure S1
**Figure** S**2**
**Figure** S**3**

## Data Availability

The data that support the findings of this study are available from the corresponding author upon reasonable request.
