# Supplementary material for: The effect of EGFR‐TKIs on survival in advanced non‐small‐cell lung cancer with EGFR mutations: A real‐world study
Source: Cancer Med. 2022 Nov 15;12(5):5630–8. doi: 10.1002/cam4.5413 (PMC10028166; doi:10.1002/cam4.5413)

Supplementary 1: Kaplan-Meier curve illustrating the OS of exon 18 vs. exon 20 =14.5 vs. undefined, P = 0.4635.


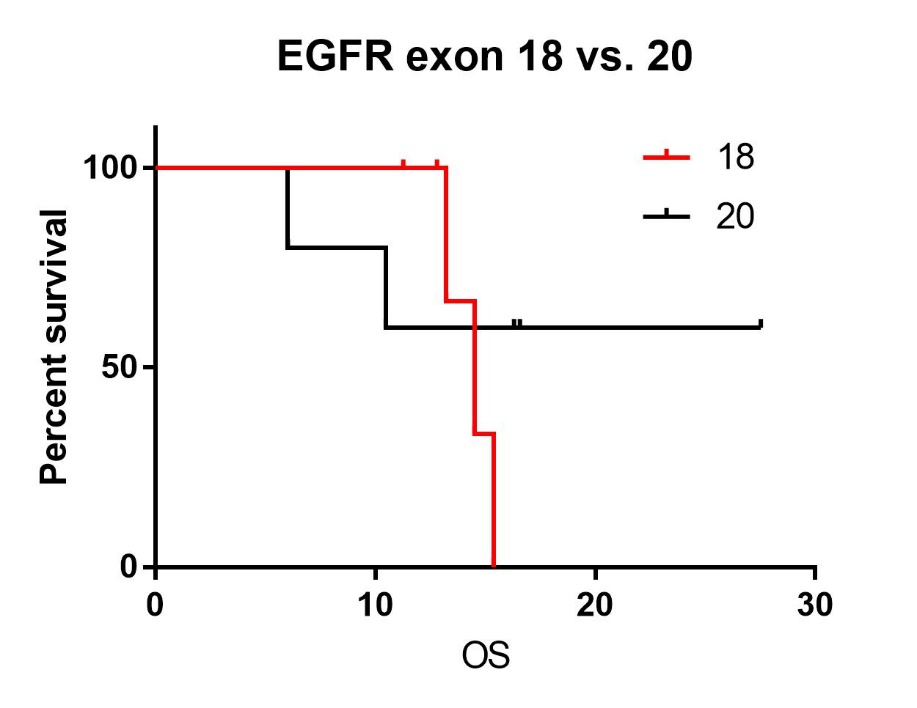


Supplementary 2: Kaplan-Meier curve illustrating the OS of patients harboring single vs. multiple EGFR mutations = 25.6 months vs. 25.3 months, P = 0.6475.


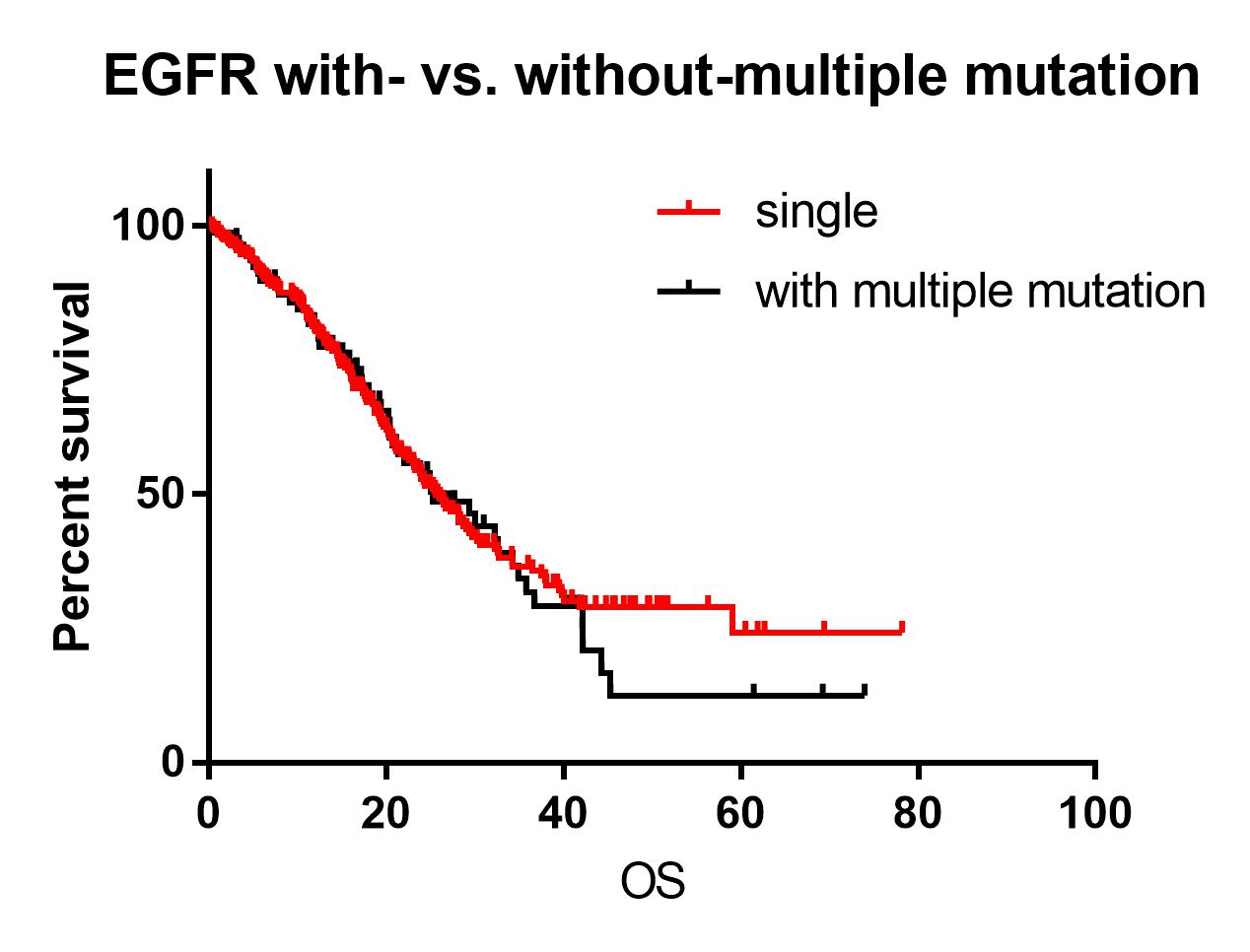


Supplementary 3: Kaplan-Meier curve illustrating the OS of patients with uncommon mutations vs. without uncommon mutations = 23.1 vs. 30.5 months, P = 0.0003.


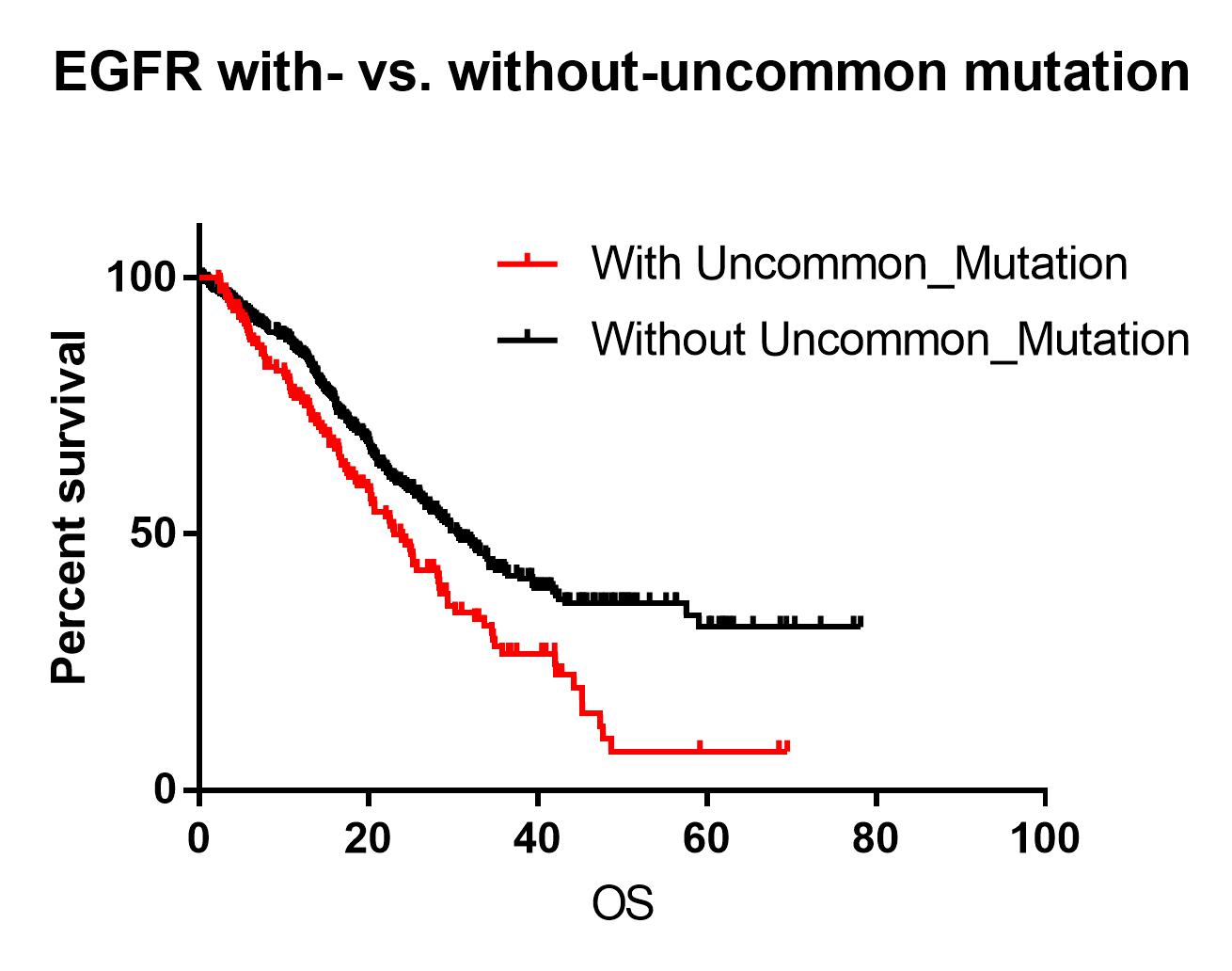

Supplement: Supplementary file 1 — Figure S1 Figure S2 Figure S3 [file CAM4-12-5630-s001.docx]
